# Analgesic effect of highly reversible ω-conotoxin FVIA on N type Ca^2+ ^channels

**DOI:** 10.1186/1744-8069-6-97

**Published:** 2010-12-21

**Authors:** Seungkyu Lee, Yoonji Kim, Seung Keun Back, Hee-Woo Choi, Ju Yeon Lee, Hyun Ho Jung, Jae Ha Ryu, Hong-Won Suh, Heung Sik Na, Hyun Jeong Kim, Hyewhon Rhim, Jae Il Kim

**Affiliations:** 1 Department of Life Science, Gwangju institute of Science and Technology, Gwangju 500-712, South Korea; 2 Department of Dental Anesthesiology, College of Dentistry, Seoul National University, Seoul 110-786, South Korea; 3 Department of Physiology, College of Medicine, Korea University, Seoul 136-705, South Korea; 4 Department of Pharmacology, Institute of Natural Medicine, College of Medicine, Hallym University, ChunCheon 200-702, South Korea; 5 Biomedical Research Center, Korea Institute of Science and Technology, Seoul 136-791, South Korea

## Abstract

**Background:**

N-type Ca^2+ ^channels (Ca_v_2.2) play an important role in the transmission of pain signals to the central nervous system. ω-Conotoxin (CTx)-MVIIA, also called ziconotide (Prialt^®^), effectively alleviates pain, without causing addiction, by blocking the pores of these channels. Unfortunately, CTx-MVIIA has a narrow therapeutic window and produces serious side effects due to the poor reversibility of its binding to the channel. It would thus be desirable to identify new analgesic blockers with binding characteristics that lead to fewer adverse side effects.

**Results:**

Here we identify a new CTx, FVIA, from the Korean *Conus Fulmen *and describe its effects on pain responses and blood pressure. The inhibitory effect of CTx-FVIA on N-type Ca^2+ ^channel currents was dose-dependent and similar to that of CTx-MVIIA. However, the two conopeptides exhibited markedly different degrees of reversibility after block. CTx-FVIA effectively and dose-dependently reduced nociceptive behavior in the formalin test and in neuropathic pain models, and reduced mechanical and thermal allodynia in the tail nerve injury rat model. CTx-FVIA (10 ng) also showed significant analgesic effects on writhing in mouse neurotransmitter- and cytokine-induced pain models, though it had no effect on acute thermal pain and interferon-γ induced pain. Interestingly, although both CTx-FVIA and CTx-MVIIA depressed arterial blood pressure immediately after administration, pressure recovered faster and to a greater degree after CTx-FVIA administration.

**Conclusions:**

The analgesic potency of CTx-FVIA and its greater reversibility could represent advantages over CTx-MVIIA for the treatment of refractory pain and contribute to the design of an analgesic with high potency and low side effects.

## Background

Voltage-gated Ca^2+ ^channels (VGCC) play an important role in the transmission of pain signals from peripheral nerves to the brain [1-3]. Of several types of VGCC, the N-type Ca^2+ ^channel is particularly important for perception of chronic nociceptive pain, as its blockade at the spinal cord and sensory neurons inhibits stimulus-evoked release of pain-inducing peptides, such as substance P, and excitatory neurotransmitters, such as glutamate [4]. It is well known that various peptide toxins from animal venom alleviate pain by specifically binding to N-type Ca^2+ ^channels with high affinity [5]. For example, several ω-conotoxins (CTxs), including GVIA, MVIIA, CVID and SO-3, exert apparent analgesic effects against both inflammatory and neuropathic pain [6-8].

Ziconotide (CTx-MVIIA; Prialt^®^) is the first CTx-derived drug to be approved for the treatment of refractory pain by the U.S. Food and Drug Administration (FDA) [9]. Not only is ziconotide a highly potent analgesic, but it induces neither drug addiction nor tolerance, as morphine does [10,11]. On the other hand, ziconotide has both cardiovascular (tachycardia and orthostatic hypotension) and nervous (confusion and dizziness) side effects [12]. These effects are currently managed by using a low initial dose that is then slowly titrated up in the clinic [12-16]; nonetheless, the drug's low clearance rate from tissues limits the extent to which side effects can be clinically managed [12,16]. A drug's clearance *in vivo *is closely related to its reversibility *in vitro*, which makes reversibility an important factor in developing analgesics targeting N-type Ca^2+ ^channels [17,18]. It was previously reported that another CTx, GVIA, could not be applied in a clinical setting because of its near irreversibility, reflecting its slow onset and offset kinetics [19]. It was also reported that CTx-CVID, a selective and relatively reversible N-type Ca^2+ ^channel blocker, has milder side effects than CTx-MVIIA [6,20,21].

**Figure 1:**
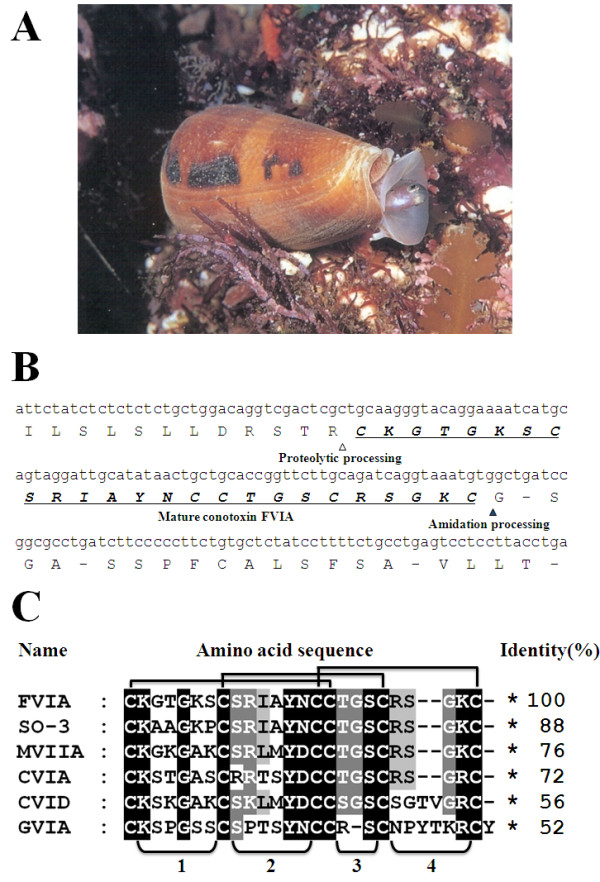
**CTx-FVIA cloned from *Conus Fulmen***. (A) *Conus Fulmen *lives in an area of subtropical sea south of Jeju island, South Korea. This piscivorous snail captures its prey mainly by paralyzing it with a mixture of toxins. (B) Nucleotide sequence and deduced amino acid sequence of CTx-FVIA. Δindicates the position at which the carboxy terminal of Arg is cleaved by conotoxin precursor proteases, and ▲ indicates the C-terminal cysteine that is thought to be amidated by monooxygenase. The mature sequence of CTx-FVIA is underlined. (C) Conotoxins homologous with CTx-FVIA are listed. Black box indicates similarity of all the peptides; dark and light gray boxes indicate similarity between five and four peptides, respectively. Six cysteines form three disulfide bonds (1st-4th, 2nd-5th, 3rd-6th) and four intercysteine loops.

In the present study, we report a new reversible CTx identified from the Korean *Conus Fulmen*, which we named CTx-FVIA. CTx-FVIA shares 76% sequence identity with CTx-MVIIA and 56% identity with CTx-CVID (Figure 1). To investigate the potency and reversibility of CTx-FVIA binding to N-type Ca^2+ ^channels, we assessed its analgesic and blood pressure effects in rodent models. Interestingly, CTx-FVIA showed a significantly higher degree of reversibility than CTx-MVIIA following blockade of human N-type Ca^2+ ^channels, despite their similar potencies. CTx-FVIA could thus represent an improvement over ziconotide for treatment of refractory neuropathic and inflammatory pain, and its study could contribute to the design of even more efficacious small-molecule analgesic agents with low side effects.

## Results

### Cloning of CTx-FVIA

To clone CTxs from *Conus Fulmen *(Figure 1A), we extracted the genomic DNA and conducted PCR using forward and reverse primers that respectively corresponded to the third intron and 3'UTR in the genomic structure of the O-superfamily [22]. This strategy was based on the fact that the O-superfamily consists of 4 exons and 3 introns, and the mature toxin is encoded in the 4th exon. Using this process, we identified a novel CTx, which we named CTx-FVIA, that is highly homologous with CTx-MVIIA, the major component of ziconotide (Prialt^®^, ELAN Corp.) [16]. The cloned nucleotide sequence is shown in Figure 1B. Sequence analysis revealed a short mature region (78 bp) encoding a peptide with a predicted size of 2566.99 Da. The residues preceding the N-terminal end of the mature peptide include basic amino acids (-XR), a putative proteolytic cleavage site, and the C-terminal end of the mature region, which is terminated with a cysteine residue that is thought to be amidated by a monooxygenase [23]. A homology search revealed that CTx-FVIA has high sequence similarity with CTxs SO3 (88%), MVIIA (76%), CVIA (72%), CVID (56%) and GVIA (52%), which all contain four loops separated by cysteine residues and show structurally similar molecular topologies, together with functionally important conserved residues (Tyr13, Lys2, Arg10 and Arg21) [24].

### Effects of CTx-FVIA and -MVIIA on human N-type Ca^2+ ^channels

**Figure 2:**
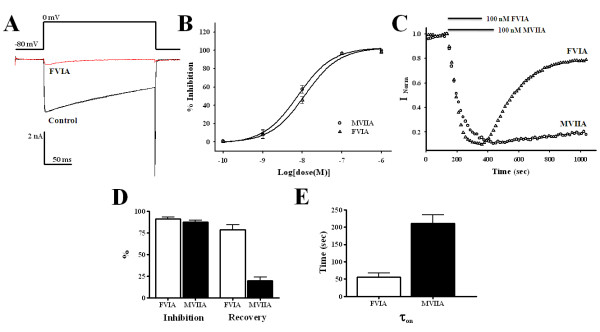
**Electrophysiological characteristics of CTx-FVIA and -MVIIA**. (A) Representative traces of N-type Ca^2+ ^currents (control) in C2D7 cells showing blockade by 100 nM CTx-FVIA. (B) Concentration-response curves for CTx-FVIA and -MVIIA (n = 3). (C) Time-course of the block induced by 100 nM CTx-FVIA or -MVIIV and its washout. Cells were held at -80 mV and depolarized to 0 mV. 100 nM CTx-FVIA (Δ) or CTx-MVIIA (○) were applied until a complete block was achieved and then washed out. Currents were measured every 15 s. (D) Potency of CTx-FVIA and -MVIIA and percent recovery after washout. (E) Averaged time constants τ_on _for CTx-FVIA and -MVIIA determined from time-course graphs fitted with a three-parameter single exponential function.

The inhibitory effects of CTx-FVIA and -MVIIA were examined on human N-type Ca^2+ ^channels stably expressed in HEK293 cells (C2D7 cells) [25]. Whole-cell Ca^2+ ^channel currents were recorded using 5 mM Ba^2+ ^as the charge carrier. Ba^2+ ^currents were evoked by 200-ms depolarizing voltage step commands from a holding potential of -80 mV. The inward Ba^2+ ^current reached a maximum amplitude with depolarization to 0 mV. Figure 2A shows that bath applications of 100 nM CTx-FVIA almost completely blocked the depolarization-activated inward Ba^2+ ^current recorded from C2D7 cells. This inhibitory effect of CTx-FVIA was dose-dependent and was similar to that of CTx-MVIIA. With both of these CTxs, the inhibitory effect plateaued at a concentration of 1 μM, and the IC_50 _values for CTx-FVIA and -MVIIA were 11.5 ± 1.4 nM and 7.96 ± 1.6 nM, respectively (Figure 2B). In other assays, CTx-FVIA had no significant activities against other subtype of Ca^2+ ^channels (T-type and P/Q-type) and TTX-sensitive Na^+ ^channels of mouse DRG neuron (data not shown), similar with the previous results of homologous CTxs [26-28].

### Reversibility of the inhibitory effects of CTx-FVIA

Figure 2C shows that N-type Ca^2+ ^currents recorded from C2D7 cells were almost completely blocked within 150-200 s after bath application of 100 nM CTx-FVIA or -MVIIA, and that the currents recovered following washout of the CTx. It is noteworthy, however, that whereas CTx-FVIA and -MVIIA showed similar abilities to block N-type Ca^2+ ^currents (91.25 ± 1.8% and 87.5 ± 1.9%, respectively, n = 5), the steady-state level to which the currents recovered was lower after washout of CTx-MVIIA (19.8 ± 3.8%) than after washout of CTx-FVIA (78.4 ± 5.8%) (Figure 2C and 2D). In addition, kinetic analyses revealed that the binding time constant (τ_on_) for CTx-MVIIA (211.4 ± 24.2 s) is much longer than that for CTx-FVIA (56.3 ± 11.2 s) (Figure 2E).

### The effect of CTx-FVIA in the formalin test

**Figure 3:**
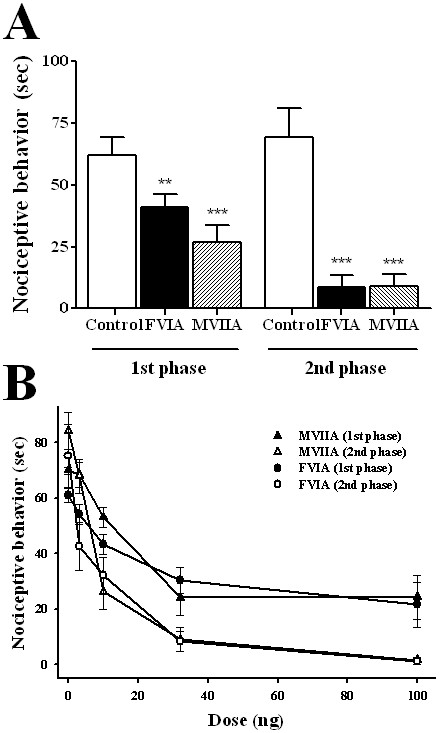
**Effects of intrathecal CTx-FVIA and -MVIIA on pain responses induced by intraplantar (left hindpaw) formalin injection**. Mice were subcutaneously injected with formalin solution (5%, 10 μl) 5 min after intrathecal administration of CTx-FVIA and -MVIIA. (A) Effect of intrathecal CTx-FVIA and -MVIIA (32 ng) on the cumulative time spent licking and biting the injected paw. Measurements were made during the first phase (0-5 min) and second phase (20-40 min) of the formalin response. There were 8-10 animals in each group. *P < 0.05, **P < 0.01, ***P < 0.005 vs. control. (B) Dose-response curves for the indicated toxins during the indicated period.

To compare the effects of CTx-FVIA and -MVIIA on nociception, we first compared their effects in the formalin test, a mouse model of nociceptive pain. Subcutaneous injection of 5% formalin into the mouse hindpaw produced acute nociceptive responses (i.e., licking, shaking and biting) during the first 5 min (first phase) after injection, as well as persistent nociceptive behavior detected during a period extending from 20-40 min after injection (second phase) [29]. Both CTx-FVIA and -MVIIA significantly reduced the cumulative time of intraplantar formalin-induced nociceptive behavior during both phases. Interestingly, both toxins were more effective during the second phase, which is closely related to inflammatory pain and central sensitization (Figure 3A) [30]. During the first phase, the ED_50 _values for intrathecal CTx-FVIA and -MVIIA were 11.7 and 13.03 ng, respectively, and the corresponding ED_50 _values in the second phase were 6.31 and 6.41 ng, respectively (Figure 3B). On the basis of the results summarized in Figure 3, we suggest that the potencies of CTx-FVIA and -MVIIA are nearly identical against both acute and persistent formalin-induced nociception. We therefore used both CTx-FVIA and -MVIIA at a dose of 10 ng for the following animal experiments.

### The effect of CTx-FVIA on acute thermal pain

**Figure 4:**
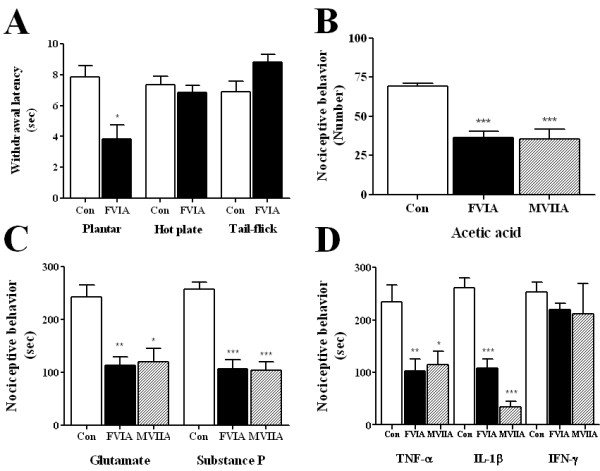
**Effects of intrathecal CTx-FVIA and -MVIIA on acute and inflammatory pains**. (A) Effects of CTx-FVIA on acute pain responses. CTx-FVIA (10 ng) was administered intrathecally 5 min before the indicated tests. There were 7-10 animals in each group. (B-D) Effects of CTx-FVIA and -MVIIA on pain responses elicited by acetic acid (intraperitoneal), substance P, glutamate, TNF-α, IL-1β and IFN-γ (intrathecal). These agents were applied 5 min after 10 ng CTx-FVIA or -MVIIA was administered intrathecally. Pain responses, including writhing, licking, biting and scratching, were measured for 30 min after injection. (B) Acetic acid-induced writhing counts. (C) Cumulative duration of neurotransmitter-evoked pain behavior. (D) Cumulative duration of inflammatory cytokine-evoked pain behavior. The data are presented as means ± SEM. There were 9-13 animals in each group. Con (control) refers to the saline-treated group. *P < 0.05, **P < 0.01, ***P < 0.005 vs. control.

To test whether CTx-FVIA has an effect on acute thermal pain, hot plate, plantar, and tail-flick tests were conducted. For the hot plate test, mice were placed on a hot plate at 54°C, and the time until they started licking or lifting their hindpaws or jumping was recorded as an indication of thermal pain. As shown in Figure 4A, there was no significant difference in the latency before paw withdrawal between mice administered intrathecal 10 ng of CTx-FVIA and those administered saline. Similarly, CTx-FVIA showed no analgesic effect in the plantar and tail-flick tests.

### The effect of CTx-FVIA in the writhing test

Intraperitoneal administration of 1% acetic acid to mice elicited a writhing response with an average score of 69.5 ± 1.9 (n = 11). Pretreatment with intrathecal 10 ng of CTx-FVIA significantly reduced the number of writhes to 36.4 ± 4.2 (n = 10). As a positive control, intrathecal CTx-MVIIA similarly reduced the number of writhes to 35.6 ± 6.1 (n = 11) (Figure 4B).

### The effect of CTx-FVIA on neurotransmitter induced pain

Intrathecal injection of substance P (0.7 μg/5 μl) or glutamate (20 μg/5 μl) produced an acute behavioral response lasting about 30 min (Figure 4C). The intrathecal administration of 10 ng of CTx-FVIA or -MVIIA significantly reduced the cumulative duration of both glutamate-induced and substance P-induced nociceptive behavior, as compared to control (Figure 4C).

### The effect of CTx-FVIA on cytokine-induced pain

Intrathecal injection of TNF-α (100 pg/5 μl), IL-1β (100 pg/5 μl) or IFN-γ (100 pg/5 μl) also produced an acute behavioral response lasting about 30 min (Figure 4D). Mice pretreated with intrathecal 10 ng of CTx-FVIA or -MVIIA showed significantly smaller pain responses to TNF-α and IL-1β than the control group. Interestingly, neither CTx-FVIA nor CTx-MVIIA had any effect on nociceptive behavior induced by IFN-γ (Figure 4D).

### The effects of CTx-FVIA on mechanical and thermal allodynia

**Figure 5:**
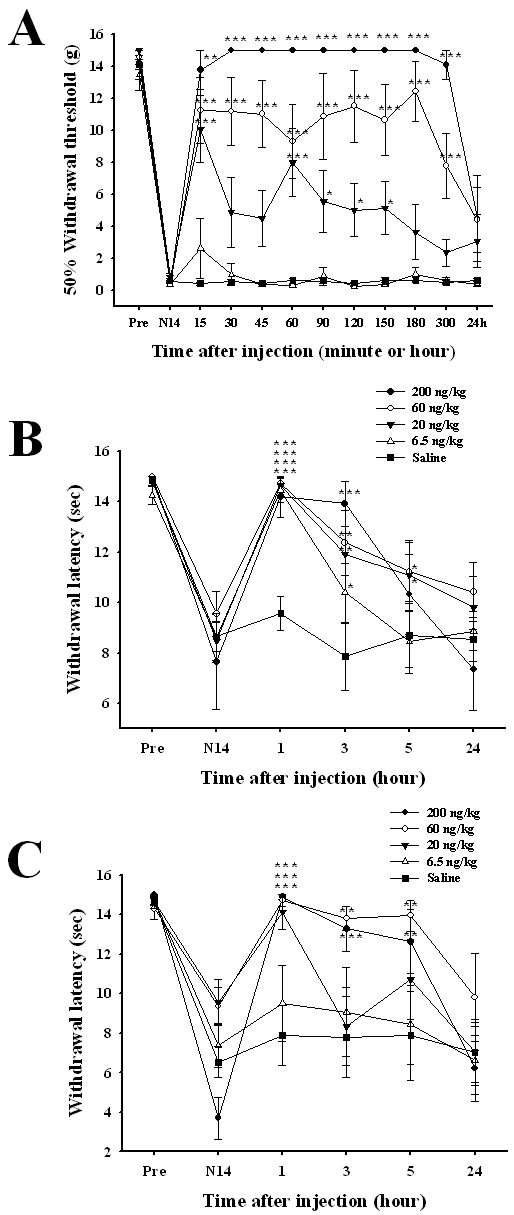
**Analgesic effects of intrathecal CTx-FVIA on neuropathic pain**. (A-C) Time-course of antinociceptive effects of intrathecal CTx-FVIA (6.5, 20, 65 and 200 ng/kg) on allodynia in an TNI rat model: (A) mechanical allodynia; (B) cold allodynia; and (C) warm allodynia. The data are presented as means ± SEM. There were 5-8 animals in each group. *P < 0.05, **P < 0.01, ***P < 0.001 vs. the saline group (two-way ANOVA followed by Bonferroni's tests).

We next investigated the effects of CTx-FVIA on mechanical allodynia in the form of neuropathic pain using an tail nerve injury (TNI) rat model in which the left inferior caudal trunk was isolated, and the spinal nerve between S1 and S2 was resected [31]. Two weeks after the injury, the rats showed symptoms of neuropathic pain, including mechanical, cold and warm allodynia. The threshold for tail withdrawal in response to stimulation with a von Frey filament was used to assess mechanical allodynia. As shown in Figure 5A, the threshold pressure for tail withdrawal was significantly reduced from 14.1 ± 0.9 g (n = 7) to 0.6 ± 0.2 g (n = 7) following the nerve injury. The effect of intrathecal CTx-FVIA was dose-dependent (ED_50 _= 43.43 ng/kg), and when rats administered a high dose (200 ng/kg), a maximal increase in the pressure threshold (15 g, n = 7, *P *< 0.001) was observed 15 min after injection, and the effect lasted for more than 5 h (Figure 5A). Thus CTx-FVIA exerts a potent analgesic effect against mechanical allodynia.

To investigate the effects of CTx-FVIA on thermal allodynia, we measured the latency of tail withdrawal in response to stimulation with warm (40°C) or cold (4°C) water. As shown in Figure 5B and 5C, TNI rats developed significant cold and warm allodynia; tail withdrawal latency was significantly reduced from 14.8 ± 0.2 sec (n = 7) to 8.3 ± 0.6 for cold allodynia and from 14.5 ± 0.5 sec (n = 7) to 6.8 ± 1.2 for warm allodynia. One hour after intrathecal administration of CTx-FVIA, tail withdrawal latency had increased in both the cold and warm thermal allodynia tests. It is noteworthy that administration of a low dose (6.5 ng/kg) of CTx-FVIA increased withdrawal latency only for cold allodynia, suggesting CTx-FVIA is more effective against cold than warm allodynia induced by tail nerve injury.

### The effect of CTx-FVIA on blood pressure

**Figure 6:**
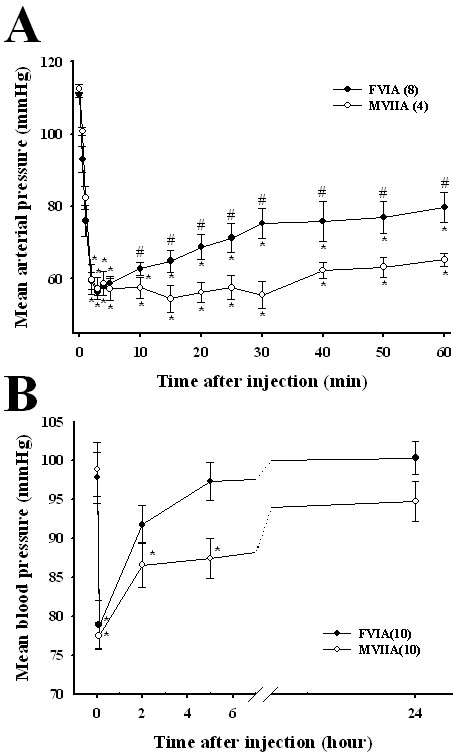
**Effects of intravenous CTx-FVIA and -MVIIA on blood pressure**. (A) Effects of intravenous CTx-FVIA and -MVIIA (100 μg/kg) on mean arterial pressure measured in the femoral artery of anaesthetized rats and (B) on mean blood pressure measured using the tail-cuff method. The data are presented as means ± SEM. *P < 0.01 vs. the pre-dosing value (repeated measures ANOVA followed by Dunnett's test). #P < 0.05 CTx-FVIA vs. CTx-MVIIA (10 min to 60 min, repeated measures ANOVA).

N-type Ca^2+ ^channels are known to be distributed in autonomic nerves, raising the possibility that intravenous injection of CTx could have adverse effects on cardiovascular function [32]. To compare the cardiovascular effects of CTx-FVIA and -MVIIA, we initially measured mean blood pressure in the femoral arteries of rats. As shown in Figure 6A, intravenous administration of 100 μg/kg CTx-FVIA or -MVIIA reduced mean arterial pressure to 56.4 ± 1.0 and 57.3 ± 3.1 mmHg, respectively, within 3 min after injection. Blood pressure then gradually recovered for the remainder of the 60-min observation period. Within 10 min, however, the recovery following CTx-FVIA injection was significantly more pronounced than after CTx-MVIIA injection (*P *< 0.05), and after 60 min blood pressures had recovered to 79.7 ± 4.1 and 65.3 ± 1.9 mmHg, respectively. In order to resolve these effects over longer periods, blood pressures were also measured using tail-cuff methods. As shown in Figure 6B, 5 min after intravenous administration of CTx-FVIA, mean blood pressure had declined significantly to 78.9 ± 3.1 mmHg from a baseline of 97.8 ± 3.3 mmHg, but the pressure nearly recovered within 5 h. By comparison, CTx-MVIIA similarly lowered mean blood pressure to 77.5 ± 1.7 mmHg within 5 min after drug injection, but then only recovered to 87.4 ± 2.6 mmHg after 5 h (P < 0.05), reflecting the poorer reversibility of the N-type Ca^2+^ channel block by CTx-MVIIA. Thus CTx-FVIA appears to exert milder cardiovascular side effects than CTx-MVIIA.

## Discussion

### Highly reversible CTx-FVIA

Cone snails, a venomous marine gastropod, uses peptide toxins (conotoxins) to capture prey and protect themselves [33]. It is estimated that approximately 500 species of cone snail each express 50-200 conotoxins with little overlap, thereby making up a library of >50,000 peptides. These toxins, which are usually characterized through gene cloning or biochemical purification, are highly useful in neuropharmacology, thanks to the specificity with which they bind to various ion channel subtypes [34]. CTx-MVIIA was biochemically purified from the venom of *Conus Magus *[35] and was recently approved by the U.S. FDA as an analgesic for the treatment refractory pain [9,11]. Unfortunately, side effects due to the poor reversibility of CTx-MVIIA narrow its therapeutic window. Given the extreme diversity of the CTx library, we endeavored to overcome the limitations of CTx-MVIIA by identifying a CTx with more favorable characteristics. CTx-FVIA was isolated through gene cloning from *Conus Fulmen*. As expected from its homology, the potencies and selectivity of CTx-FVIA and -MVIIA were similar, as judged from their effects on evoked N-type Ca^2+ ^currents (Figure 2B) and other Ca^2+ ^and Na^+ ^currents. Interestingly, however, following blockade by CTx-FVIA, currents showed significantly greater recovery than after blockade by CTx-MVIIA. Although our observation of the poor reversibility of CTx-MVIIA is at variance with some other reports [20,26,36], clinical observations support our finding that CTx-MVIIA is poorly reversible [12]. Our assay system may reflect the clinical results because C2D7 cells may mimic human neuropathic or inflammatory pain states by overexpressing the α_2b_δ subunits of the N-type Ca^2+ ^channel [25], which are up-regulated by neuropathic pain, and by modulating the α_1B _subunit [37,38]. In addition, comparison of their τ_on _kinetic constant suggests CTx-FVIA binds to the channels more rapidly than CTx-MVIIA (Figure 2E). We would also expect that CTx-FVIA dissociates from channels faster than CTx-MVIIA because the two toxins have similar IC_50 _values (apparent K_d _= K_off_/K_on_). Several factors reportedly affect the binding kinetics and reversibility of conotoxins. These include the holding potential (V_h_), the divalent cation concentration, the presence of the α_2_δ auxiliary subunit, and mutation of channel protein residues (Gly1326) or CTx residues (Arg10) [20,36,39-41]. Given that these factors were identical for each CTx in our setting, it seems that other factors, perhaps related to the six residues that differ between their sequences, control the binding kinetics and reversibility CTx-FVIA and -MVIIA. It would be interesting to undertake mutagenesis studies to explore how these localized differences influence the binding kinetics of these two toxins. The fast kinetics and relatively high reversibility of CTx-FVIA could be of clinical importance, as its greater reversibility could reduce its toxicity and facilitate management of its side effects [17,18].

### Analgesic effects of CTx-FVIA

The potency with which CTx-FVIA blocks N-type Ca^2+ ^channels and its homology with CTx-MVIIA suggests CTx-FVIA should be effective against pain *in vivo*. To test this hypothesis, we used several rodent pain models. Pain is classified as acute pain, which is essential for survival of the organism, and persistent pain, which is pathological and often accompanied by a lowered pain threshold. The formalin test is useful to investigate drug effects on both acute and persistent pain because it produces two phases of nociceptive behavior. First phase pain is caused by direct effects on nociceptors leading to activation of primary afferent fibers, but second phase pain is caused by tonic inflammatory nociceptive responses. The effects of CTx-FVIA and -MVIIA were more pronounced during the second phase than during the first, indicating that CTxs reduce pathological pain more effectively than acute pain (Figure 3). When an effective dose (10 ng) of CTx-FVIA was intrathecally administered in acute thermal pain models (hot plate or tail-flick), the latency times were little affected or perhaps even reduced (plantar test) (Figure 4A). By contrast, CTx-FVIA effectively reduced thermal allodynia caused by peripheral nerve injury (Figure 5B and 5C). These findings are similar to results previously reported for CTx-MVIIA in tail-flick or formalin tests [6,8,42] and suggest that N-type Ca^2+ ^channel blockers are more effective against chronic pain than acute pain. This may be due in part the fact that expression of N-type Ca^2+ ^channels is up-regulated by chronic pain, reflecting the plasticity of the affected synapses [37,38,43,44].

Chronic pain is further divided into inflammatory pain and neuropathic pain, based on the underlying mechanism [43]. Inflammatory pain is caused by the actions of various inflammatory mediators, including cytokines, growth factors, neurotransmitters and protons, while neuropathic pain is caused by nervous system dysfunction and is well characterized in rodents with respect to the location and form of the nerve injury involved [45,46]. In the present study, intraperitoneal injection of acetic acid or intrathecal injection of glutamate, substance P, TNF-α, IL-1β or IFN-γ were used to simulate inflammatory pain [47]. Intraperitoneal injection of acetic acid is considered to be a model of visceral inflammatory pain, while neurotransmitters and pro-inflammatory cytokines play important roles in the development of inflammatory pain in the spinal cord, where they produce pain signals by directly activating nociceptors or by sensitizing the peripheral nervous system [43]. At effective doses (e.g., 10 ng), CTx-FVIA and -MVIIA exerted analgesic effects in all of these inflammatory pain models, with the exception of IFN-γ-induced pain (Figure 3 and 4), which suggests the involvement of N-type Ca^2+ ^channels in the pain pathway activated by these inflammatory mediators. The ways in which inflammatory mediators increase pain sensitization through ion channel modulation have been described in other cases. For example, second phase pain in the formalin test is related to an inflammatory reaction in peripheral tissues [30] and requires nociceptive neurotransmitter release and activation of postsynaptic N-methyl-D-aspartate (NMDA) receptors [48]. Intrathecal neurotransmitters and pro-inflammatory cytokines also induce inflammatory pain by modulating ion channels [49-51]. In particular, TNF-α and IL-1β increase tetrodotoxin-resistant Na^+ ^channel currents and sensitize N-type Ca^2+ ^channels [52,53]. It is noteworthy that CTx-FVIA and -MVIIA do not reduce IFN-γ induced pain, although they effectively alleviate pain induced by acetic acid, substance P, glutamate, TNF-α or IL-1β (Figure 4B, C and 4D). It may be that different nociceptive stimuli use different pain pathways or modulate different channels [43,54]. It is not well understood how substance P, glutamate and IL-1β affect N-type Ca^2+ ^channels, but our results suggest they at least evoke pain signals via N-type Ca^2+ ^channels, probably situated postsynaptically. In two neuropathic pain models, SNL (data not shown) and TNI, CTx-FVIA also exerted dose- and time-dependent anti-allodynic effects (Figure 5). These effects of N-type Ca^2+ ^channel blockers have been observed in several neuropathic pain models [6,55,56], and our results showing that CTx-FVIA is effective against neuropathic pain developed by S1/S2 nerve injury are similar. Taken together, our results indicate that the CTx-FVIA is as effective an analgesic as CTx-MVIIA in inflammatory and neuropathic pain models, and establishes that the residues essential for blocking N-type Ca^2+ ^channels are effective on a scaffold of CTx-FVIA (Figure 1C) [24,57-59].

### In vivo reversibility of CTx-FVIA

N-type Ca^2+ ^channels are widely expressed in autonomic nerves innervating the blood vessels, as well as presynaptic nerves in the spinal cord and brain [32,60]. Activation of N-type Ca^2+ ^channels in sympathetic nerves leads to release of norepinephrine, thereby increasing blood pressure. Consequently, the cardiovascular side effects of CTxs would be expected to reflect inhibition of norepinephrine release. For example, intravenous injection of CTx-GVIA, -MVIIA or -CVID induces hypotension and reflex tachycardia [60]. To assess the cardiovascular effects of CTx-FVIA and -MVIIA, we measured their effects on mean arterial blood pressure with a dose of 100 μg/kg. The CTx dose (100 μg/kg) is very high. To visualize a recovery pattern with reversible effects, however, we used a high dosage of the toxin (100 μg/kg). When a relatively low CTx dose (10 μg/kg) was intravenously administered into rats, in fact, we could not observe any apparent difference between CTx-FVIA and CTx-MVIIA in recovery pattern of blood pressure. Similarly it was reported that Wright et al. used a high dose of toxin (100 μg/kg) in an experiment for cardiovascular and autonomic effects [60]. As with its effect on N-type Ca^2+ ^currents, the effect of CTx-FVIA on blood pressure was more reversible than that of CTx-MVIIA (Figure 2C and Figure 6). Although slow reversibility could be an advantage, in that it could prolong the therapeutic effects of CTx-MVIIA, it also makes management of the side effects caused by prolonged N-type Ca^2+ ^channel blockade difficult. Consequently the greater reversibility of CTx-FVIA could be advantageous for its clinical application in the treatment of pathological pain [17,18,61].

## Conclusions

Collectively then, our findings suggest that CTx-FVIA could represent an improvement over CTx-MVIIA for treatment of refractory neuropathic and inflammatory pain. Additional study will be required to fully understand the factors contributing to the reversibility of CTx-FVIA, which we would expect to facilitate improvement in the clinical applicability and efficacy of this class of drugs.

## Methods

### Cloning of CTx-FVIA

Specimens of *Conus Fulmen *were collected from an area of subtropical sea south of Jeju island, Republic of Korea. Frozen hepatic tissue (0.1 g) from *Conus Fulmen *was added to 1 ml of GES reagent (5 M guanidinium thiocyanate, 100 mM EDTA, 0.5% v/v Sarkosyl) and then ground with a glass homogenizer [62]. After centrifugation, the supernatant was mixed with phenol and chloroform and centrifuged again at room temperature. The colorless upper aqueous phase was then transferred to a fresh tube and mixed with 1 ml of isopropanol to precipitate the genomic DNA. The resultant pellet was washed twice in 70% ethanol and dissolved in 50 μl of 10 mM Tris-HCl (pH 8.5). The collected genomic DNA was used as a template for PCR with a forward primer (5'-CTCTCTCTCTCTCTGCTGGAC-3') and reverse primer (5'-CAGAAAAGGATAGAGCACAGAAGG-3') corresponding respectively to the third intron and 3' UTR in the genomic structure of the O-superfamily. The PCR protocol entailed 35 cycles of 94°C for 30 s, 55°C for 30 s and 70°C for 45 s. The purified PCR products were ligated into pGEM-Teasy vector (Promega) and then transformed to competent DH5α cells. The nucleic acid sequences of the expressed clones were determined using an ABI Prism 3700 DNA analyzer (Figure 1B).

### Peptide synthesis

The sequences of CTx-FVIA and CTx-MVIIA are shown in Figure 1C. The linear precursors of each peptide were synthesized using solid-phase methodology with Fmoc chemistry, starting from Rink-amide resin. Briefly, synthetic peptides were deprotected and cleaved using a mixture containing 82.5% TFA, 2.5% 1,2-ethanedithiol, 5% H_2_O, 5% thioanisole and 5% phenol (v/v). Each linear peptide was diluted to a final concentration of 2.5 × 10^-5 ^M and subjected to oxidative disulfide bond formation for 3 days at 4°C in 0.3 M ammonium acetate buffer (pH 7.8) containing 0.5 M guanidine hydrochloride and reduced/oxidized glutathione (peptide:GSH:GSSG molar ratio was 1:100:10). Preparative RP-HPLC was performed using a Shimadzu LC-6AD system with an ODS column (20 × 250 mm). The purity of the synthetic peptides was confirmed by analytical RP-HPLC and MALDI-TOF mass spectrometry.

### Electrophysiological measurements

C2D7 cells expressing the α_1B-1_, α_2b_δ, and β_1b _subunits of N-type Ca^2+ ^channels were cultured in DMEM medium supplemented with 1% penicillin/streptomycin, 5% bovine calf serum, 0.5 g/L geneticin and 30 U/ml hygromycin [25]. Total Ba^2+ ^currents were measured using the whole-cell patch clamp technique. Coverslips with cells were mounted in a perfusion chamber and constantly perfused using a gravity feed system with a modified HEPES-balanced external solution (151 mM tetraethylammonium chloride, 10 mM HEPES, 5 mM BaCl_2_, 1 mM MgCl_2_, and 10 mM glucose; pH was adjusted to 7.4 and osmolarity to 310 mOsm) to isolate the Ba^2+ ^currents. Electrodes were made of borosilicate glass and had a resistance of 3-4 MΩ when filled with pipette solution. The pipette solution contained 100 mM CsCl, 1 mM MgCl_2_, 10 mM HEPES, 10 mM BAPTA, 3.6 mM MgATP, 14 mM phosphocreatine (CrP), 0.1 mM LiGTP and 50 U/ml creatine phosphokinase (CrPK). The solution pH was adjusted to 7.2 using CsOH. Ba^2+ ^currents were recorded using an EPC-9 amplifier, and the data were analyzed using Pulse/Pulsefit (HEKA, Germany) and GraphPad Prism (GraphPad Inc.) software.

### Drugs and animals

Formalin, acetic acid, substance P and glutamate were purchased from Sigma Chemical Co. (St. Louis, MO). TNF-α, IFN-γ and IL-1β were from R and D Systems Inc. (Minneapolis, MN, USA). All drugs were prepared just before use in 0.9% NaCl (w/v). Adult male Sprague-Dawley rats weighing 150-200 g or ICR mice weighing 23-25 g were used. Animals were housed together in groups in a room maintained at 22 ± 0.5°C and had unrestricted access to food and water. The animals were allowed to adapt to the experimental conditions in the laboratory for at least 2 h before pain testing. All procedures were conducted in accordance with the Guide for Care and Use of Laboratory Animals published by the U.S. National Institutes of Health and the ethical guidelines of the International Association for the Study of Pain.

### Intrathecal and intravenous injection of CTx-FVIA and CTx-MVIIA

Using the method of Hylden and Wilcox [63,64], conscious mice or enflurane-anesthetized rats were intrathecally injected using a 30-gauge needle connected to a 25-μl Hamilton syringe via polyethylene tubing. The injected volume was 5 μl, and the injection site was verified by injecting a similar volume of 1% methylene blue solution and determining the distribution of the dye in the spinal cord. The injected dye was distributed both rostrally and caudally but diffused only a short distance (about 0.5 cm), and no dye was found in the brain. Before any experiments were done, the success rate for intrathecal injections was consistently >95%. Intravenous injections were made into the lateral tail vein of conscious rats using a 26-gauge needle connected to 1 ml syringe.

### Formalin test

Formalin tests were carried out as previously described by Hunskaar et al. [29]. Briefly, mice were intrathecally injected with 3.2, 10, 32, or 100 ng/5 μl of CTx-FVIA or CTx-MVIIA, or physiological saline (control) 5 min before formalin testing. Thereafter, 10 μl of 5% formalin in saline (0.9% NaCl) were subcutaneously injected into the plantar surface of the left hindpaw, and the elicited behaviors, including licking, biting, scratching or shaking, were recorded and counted. Throughout this experiment the mice were observed in a transparent observation chamber (acrylic-plastic, 20 × 20 cm in diameter × height), and the counts were divided into two phases. The first phase extended from 0-5 min after injection, while the second phase extended from 20-40 min after injection. The total observation period was 40 min.

### Acute thermal pain tests

Mice were intrathecally injected with 5 μl of CTx-FVIA (0.01 μg/5 μl) or physiological saline (control) 5 min before testing. The tail-flick test was then performed using a commercially available apparatus (Model TF6, EMDIE Instrument Co.). The body of each mouse was held with a hand, and their tail was extended so that the distal part could be irradiated with light at an intensity of 3.8 mWatt/cm^2 ^to impose a heat stimulus. The time it took the mouse to flick its tail was then recorded with a cut-off time of 10 sec. Plantar test of pain threshold was also performed using a commercially available apparatus (Plantar test 7371, Ugo Basile). In this case, each mouse was subjected to a heat stimulus by irradiating the plantar pad of one paw with light at an intensity of 90 mWatt/cm^2^, and the latency of paw withdrawal was measured with a cut-off time of 15 sec. For the hot-plate test, mice were individually placed on a hot plate (54°C) and the reaction time, from placement of the mouse on the hot plate to the time the mouse began licking a front-paw was measured with a cut-off time of 30 sec. The dimensions of the hot plate apparatus were 30 × 30 × 30 cm (Model 39 Hot Plate, Itic Life Science).

### Writhing test

Mice were intrathecally injected with 0.01 μg/5 μl of CTx-FVIA, -MVIIA, or physiological saline (control) 5 min before testing. For the writhing test, mice were intraperitoneally injected with 0.5 ml of 1% acetic acid in saline. The number of writhes was counted during a 30 min period following the injection. A writhe was defined as a contraction of the abdominal muscles accompanied by an extension of the forelimbs and elongation of the body.

### Nociceptive behavior induced by substance P, glutamate or inflammatory cytokines

Mice were intrathecally injected with 0.01 μg/5 μl of CTx-FVIA, -MVIIA, or physiological saline (control) 5 min before testing. Thereafter, mice were intrathecally injected with 0.7 μg/5 μl of substance P, 20 μg/5 μl of glutamate or 100 pg/5 μl of TNF-α, IFN-γ or IL-1β and then immediately placed in an observation chamber (described above). The durations of the pain-like responses, which included licking, biting and scratching the abdomen and hind regions of the body, were recorded for 30 min. The cumulative durations of the pain-like responses were measured using a stopwatch. These characteristic behaviors induced by the pharmacological effects of the injected agents were not observed in the vehicle-treated control group.

### Tail nerve injury (TNI) rat models

Under enflurane anesthesia, the left superior and inferior caudal trunks were isolated and resected between the S1 and S2 spinal nerves [31,65]. To prevent possible rejoining of the proximal and distal ends of the severed trunk, a piece of the trunk (about 1-2 mm) was removed from the proximal end. Two weeks after surgical procedure, the mechanical sensitivity of the tail was measured based on the frequency of tail flicks induced by application of different von Frey filaments. Each rat was placed in a transparent plastic tube, 5.5 × 15 cm or 6.5 × 18 cm (diameter × length), depending on their body size; the tube had to be small enough to prevent the rat from turning around. For testing, a series of eight von Frey filaments (0.41, 0.70, 1.20, 2.00, 3.63, 5.50, 8.50 and 15.14 g) were used after calibration. The von Frey filaments were applied to the tails, starting with a filament of 2.00 g; initially the most sensitive area was determined by rubbing various areas of the tail with the shank of the von Frey hair, and then, this area was challenged systematically by applying static pressure with the von Frey hair to locate the spot. An abrupt tail movement of about 0.5-20 cm in response to von Frey hair stimulation is considered to be an abnormal response, indicative of mechanical allodynia. During repeated trials, the test stimuli can be easily delivered to the same spot without difficulty, since the tail is usually stationary. Rats were intrathecally injected with 6.5, 20, 65, or 200 ng/kg of CTx-FVIA, or physiological saline (control). The 50% withdrawal threshold was determined using the up-down method described by Chaplan et al. [66].

Thermal sensitivity was assessed by immersing the tail in cold (4°C) or warm (40°C) water. Following tail immersion, the investigator observed the tail to see if it moved abruptly, as in the tail-flick test, and measured the latency of the tail response with a cut-off time of 15 s. The tail immersion test was repeated five times at 5 min intervals to obtain the average tail response latency for each animal on each experimental day.

### Mean arterial pressure test

All surgical procedures were performed using aseptic technique. A polyethylene-10 catheter was inserted into the femoral artery of Sprague-Dawley rats to record invasive arterial blood pressure (IntelliVue MP30, Philips). Catheterization and all of the measurement for blood pressure were done under general anesthesia with isoflurane. After taking the basal level of mean arterial pressure, 100 μg/kg of CTx-FVIA or -MVIIA was injected intravenously, and mean arterial pressure was measured over time.

### Longer-term monitoring of blood pressure using a tail-cuff

Twenty Sprague-Dawley rats were randomly divided into two groups receiving CTx-FVIA (100 μg/kg) or CTx-MVIIA (100 μg/kg). Each group included 10 rats, and drugs were administered by intravenous injection. The systolic and diastolic blood pressures were measured before and 5 min, 3 h, 5 h and 24 h after drug administration using the tail-cuff method [67].

### Statistical analysis

Data are presented as means ± SEM, and ED_50 _values are reported as geometric means accompanied by their respective 95% confidence limits. The statistical significance of differences between groups was assessed with *t*-tests (and nonparametric tests) or with one-way or two-way analysis of variance (ANOVA) followed by Bonferroni's or Dunnett's post-hoc test. All tests were performed using GraphPad Prism version 4.0 for Windows. Values of *P *< 0.05 were considered significant.

## List of abbreviations

HEK: human embryonic kidney; IC_50_: half maximal inhibitory concentration; TTX: tetrodotoxin; DRG: dorsal root ganglion; ED_50_: half maximal effective dose; IFN-γ: interferon-γ; IL-1β: interleukin-1β; TNF-α: tumor necrosis factor-α; SNL: spinal nerve ligation; TFA: trifluoroacetic acid; DMEM: dulbecco's modified eagle medium; BAPTA: 1,2-bis(o-aminophenoxy)ethane-N,N,N',N'-tetraacetic acid; ATP: adenosine-5'-triphosphate; SEM: standard error of the mean

## Competing interests

The authors declare that they have no competing interests.

## Authors' contributions

SL and JIK initiated and designed this project, analyzed data and drafted the manuscript. SL and JHR synthesized CTx-FVIA and CTx-MVIIA. YK and HR conducted electrophysiological experiments. SKB, HWC, HWS, HSN, and HJK conducted animal studies. JYL and HHJ participated in experimental design, data analysis and the finalization of the manuscript. All authors have read and approved the final manuscript.
